# Rapid and transient evolution of local adaptation to seasonal host fruits in an invasive pest fly

**DOI:** 10.1002/evl3.304

**Published:** 2022-12-08

**Authors:** Laure Olazcuaga, Julien Foucaud, Candice Deschamps, Anne Loiseau, Jean‐Loup Claret, Romain Vedovato, Robin Guilhot, Cyril Sévely, Mathieu Gautier, Ruth A. Hufbauer, Nicolas O. Rode, Arnaud Estoup

**Affiliations:** ^1^ CBGP, INRAE, CIRAD, IRD, Institut Agro, Univ Montpellier Montpellier 34988 France; ^2^ Department of Agricultural Biology Colorado State University Fort Collins Colorado 80523 USA; ^3^ Chambre d'agriculture de l'Hérault Lattes 34875 France; ^4^ Graduate Degree Program in Ecology Colorado State University Fort Collins Colorado 80523 USA

**Keywords:** Adaptive phenotypic plasticity, *Drosophila suzukii*, genetic effects, host fruits, local adaptation, reciprocal common garden experiment

## Abstract

Both local adaptation and adaptive phenotypic plasticity can influence the match between phenotypic traits and local environmental conditions. Theory predicts that environments stable for multiple generations promote local adaptation, whereas highly heterogeneous environments favor adaptive phenotypic plasticity. However, when environments have periods of stability mixed with heterogeneity, the relative importance of local adaptation and adaptive phenotypic plasticity is unclear. Here, we used *Drosophila suzukii* as a model system to evaluate the relative influence of genetic and plastic effects on the match of populations to environments with periods of stability from three to four generations. This invasive pest insect can develop within different fruits, and persists throughout the year in a given location on a succession of distinct host fruits, each one being available for only a few generations. Using reciprocal common environment experiments of natural *D. suzukii* populations collected from cherry, strawberry, and blackberry, we found that both oviposition preference and offspring performance were higher on medium made with the fruit from which the population originated than on media made with alternative fruits. This pattern, which remained after two generations in the laboratory, was analyzed using a statistical method we developed to quantify the contributions of local adaptation and adaptive plasticity in determining fitness. Altogether, we found that genetic effects (local adaptation) dominate over plastic effects (adaptive phenotypic plasticity). Our study demonstrates that spatially and temporally variable selection does not prevent the rapid evolution of local adaptation in natural populations. The speed and strength of adaptation may be facilitated by several mechanisms including a large effective population size and strong selective pressures imposed by host plants.

**Impact Summary**

Natural populations often exhibit good “fit” to the environment they are in. However, environments change over time and space, and following change, the fit between a population and its environment may be poor. A question of long‐standing interest is how do populations track changing environments to maintain performance? Two main mechanisms are known: (*i*) genetic shifts in the form of local adaptation, in which traits evolve over time through differences in performance of individuals harboring different genetic variants, and (*ii*) plastic shifts, or adaptive phenotypic plasticity, in which traits immediately change in response to environmental change. Adaptation is common when environments change over multiple generations, whereas plasticity is common when environments change over an individual's lifetime. However, it remains unclear whether plasticity or adaptation is more vital to maintaining performance when environments change at an intermediate pace. *Drosophila suzukii* is well‐suited to evaluating the relative importance of plasticity and adaptation in response to an intermediate pace of environmental change. This invasive pest species experiences an environment that shifts every one to four generations as host fruits change over time and space. Here, we studied natural populations of *D. suzukii* collected from different hosts. Using reciprocal common environment experiments, we evaluated their performance on their source and alternative hosts. *Drosophila suzukii* populations were most fit on their source host, successfully tracking an intermediate pace of environmental change. We developed a statistical method to quantify the contributions of adaptive plasticity and local adaptation in determining performance. We found that performance was mainly maintained through local adaptation to each new host in succession. This study highlights that spatially and temporally variable selection does not prevent local adaptation and, on the contrary, illustrates how rapid the adaptive process can be. It also provides a novel statistical tool that can be applied to other systems.

Adaptation is the process whereby organisms come to match environmental conditions in ways that enhance fitness. When selection differs between environments, adaptation to the home environment may result in reduced fitness in other environments (e.g., Via, 1991; Kawecki and Ebert 2004; Torres Dowdall et al. 2012). Such a pattern of higher fitness in the natal environment than in alternate environments is typically understood to be due to local adaptation of genetically differentiated populations. However, this pattern can also arise through adaptive phenotypic plasticity (Torres Dowdall et al. 2012; Yampolsky et al. 2014; Rago et al. 2019; Bonnet et al. 2021; Enbody et al. 2021), which enables populations to rapidly track environmental change without genetic change, through shifts in behavior and development, or parental and transgenerational effects (Price et al. 2003).

Adaptive phenotypic plasticity can modify phenotypes more rapidly than adaptation from standing genetic variation (Levins 1968; Gillespie 1974; Botero et al. 2015; Tufto 2015). It is expected to evolve when environmental change is frequent, occurring within a generation, and can be assessed through reliable cues such as changing light regimes predicting oncoming colds (Gavrilets and Scheiner 1993; Jong 1999; Tufto 2000). However, plasticity may be costly, in that it requires energy to alter phenotypes, in addition to the material expenses involved in sensory and regulatory machinery (Dewitt et al. 1998; Van Buskirk and Steiner 2009; Auld et al. 2010). Given such potential costs, when environments change more slowly, that is, over the course of multiple generations, genetic differentiation leading to local adaptation is favored by selection (Levins 1968).

Many environments change at an intermediate frequency, and are not necessarily predictable. Population responses to such change could be shaped by genetic adaptation, phenotypic plasticity, or both (Schmid 1992). Here, we seek to understand the relative contribution of local adaptation and adaptive plasticity in maintaining fitness of populations living in anthropogenically altered environments, specifically in agricultural areas, that change at an intermediate frequency.

Local adaptation can be distinguished from adaptive phenotypic plasticity by performing reciprocal common environment experiments, in which the performance of populations in their original environment as well as in other environments is measured over several generations (Fig. 1A vs. 1B). The relative importance of genetic and plastic responses in matching the phenotype of natural populations to their environment is just beginning to be studied in cases with frequent or infrequent environmental changes (Rago et al. 2019; Bonnet et al. 2021; Enbody et al. 2021), or in seasonal changes (Stone et al., 2019). However, to our knowledge, no study has investigated the importance of local adaptation and phenotypic plasticity when populations evolve in heterogeneous environments with periods of environmental stability from three to four generations. Three main experimental and statistical reasons account for an overall lack of empirical evidence regarding the relative importance of genetic and plastic responses in this situation. First, selective pressures are hard to control *in natura*, as they can vary both spatially and temporally (Rausher 1988; Fry 1996; Hansen et al. 2006; Barghi et al. 2020). This issue is best tackled by performing reciprocal common environment experiments in the laboratory or other controlled environment where local adaptation to a given biotic or abiotic factor of interest can be tested on populations from different geographical locations (Fig. 1A; Turesson 1922; Kawecki and Ebert 2004; Hereford 2009). Second, local adaptation can be masked by variation in performance among populations due to environmental effects (non‐adaptive phenotypic plasticity; Fig. 1C). Indeed, local adaptation can only be distinguished from non‐adaptive and adaptive phenotypic plasticity by performing reciprocal common environment experiments. In these experiments, individuals from populations from different geographical locations are raised in the same environment over one or several generations (Kawecki and Ebert 2004). Third, although multigeneration laboratory experiments allow estimates of both genetic and plastic responses (Merila and Hendry 2014), reliable statistical tools to quantify and test their relative contribution to the performance of populations across environments are currently lacking.

**Figure 1:**
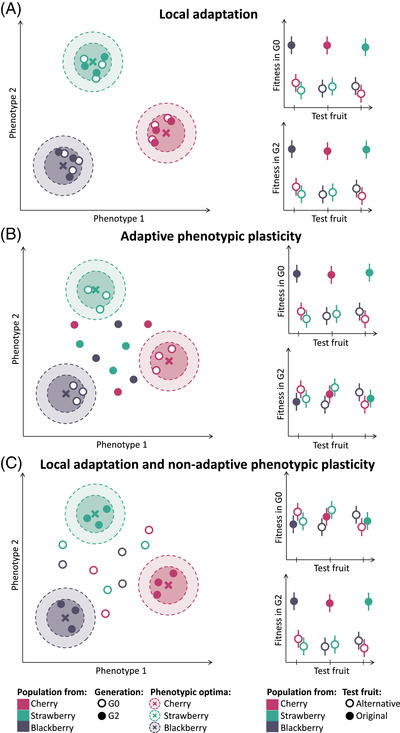
Theoretical predictions for (A) local adaptation, (B) adaptive phenotypic plasticity, and (C) local adaptation with non‐adaptive phenotypic plasticity. In the left panels, the two‐dimensional fitness landscapes are represented. For each fruit, the position of the phenotypic optimum providing maximal fitness is represented by a cross. The positions of populations in G0 are represented by open and G2 by closed circles. The right panels provide the fitness measures in G0 or G2 in the original and in alternative fruits (closed and open circles, respectively).

Phytophagous insects are valuable biological models to investigate the relative contribution of genetic and plastic responses to varying natural environmental conditions. The host plants of these insects represent heterogeneous resources that vary spatially and temporally in both availability and quality. Oviposition preference for and offspring performance on a host plant are important fitness components that shape host use and specialization in phytophagous insects (Jaenike 1978; Ravigné et al. 2009) and can either be plastic (e.g., learned preference for the habitat encountered as offspring; Dury and Wade 2020) or genetic (e.g., preference of individuals for the environment they are the best adapted to; Ravigné et al. 2009). When hosts co‐occur and are present in the same location at the same time, phytophagous insects may maximize their fitness by choosing the best host (e.g., best quality; Jaenike 1978). However, the host distribution of most phytophagous insects is often spatially or temporally heterogeneous (Denno and Dingle 1981). Under this scenario, we expect the preference for the host of origin to evolve, as searching for a better host may involves numerous costs (e.g., increased predation risk, increased energetic expenditure, increased risk of inbreeding due to low population size; Papaj and Prokopy 1989; Davis and Stamps 2004). In the present study, we focused on the spotted wing drosophila, *Drosophila suzukii*, a generalist crop pest with a broad range of host fruits (Lee et al. 2011). This invasive insect species persists throughout the growing season on a succession of different host species as they become available (Kenis et al. 2016). Hence, wild populations of *D. suzukii* evolve in a changing environment. Given the seasonality of the fruits and the development time of the insect (Burrack et al. 2013; Poyet et al. 2015; Aly 2018), they likely spend only three or four generations on a given host fruit. In contrast to most other generalist *Drosophila* species, the hosts used by *D. suzukii* are well known (Lee et al. 2011; Walsh et al. 2011). Hence, wild *D. suzukii* metapopulations represent tractable systems to experimentally estimate the relative contributions of genetic and plastic effects to phenotypic adaptation.

To characterize phenotypic adaptation in this species, we sampled natural populations on different fruits and performed a reciprocal common environment experiment over multiple generations in the laboratory. We tested for a pattern of higher performance on or oviposition preference for the fruit they were sampled from, relative to alternative fruits. To do this, we developed a statistical method to estimate the relative contributions of genetic and plastic effects in phenotypic adaptation. Additionally, we examined if oviposition preference and offspring performance traits were correlated, which could speed their co‐evolution (Jaenike 1978).

## Material and Methods

### POPULATION SAMPLING AND LABORATORY MAINTENANCE

We investigated the adaptation of natural *D. suzukii* populations to three host plants: *Prunus avium* (cherry), *Fragaria* × *ananassa* Duch (strawberry), and *Rubus fruticosus* (blackberry). We chose these fruit species to represent agronomically important crops (cherry, strawberry) and a wild host fruit considered to be an important reservoir (blackberry; Lee et al. 2011; Poyet et al. 2015). In the Northern hemisphere, most varieties of cherry and strawberry are available mainly from the end of the spring to the end of the summer, whereas blackberry is mostly available from the middle of the summer to the middle of the fall. These three species cover the main active period of *D. suzukii* in the Northern hemisphere (i.e., from May to October; Walsh et al. 2011).

**Figure 2:**
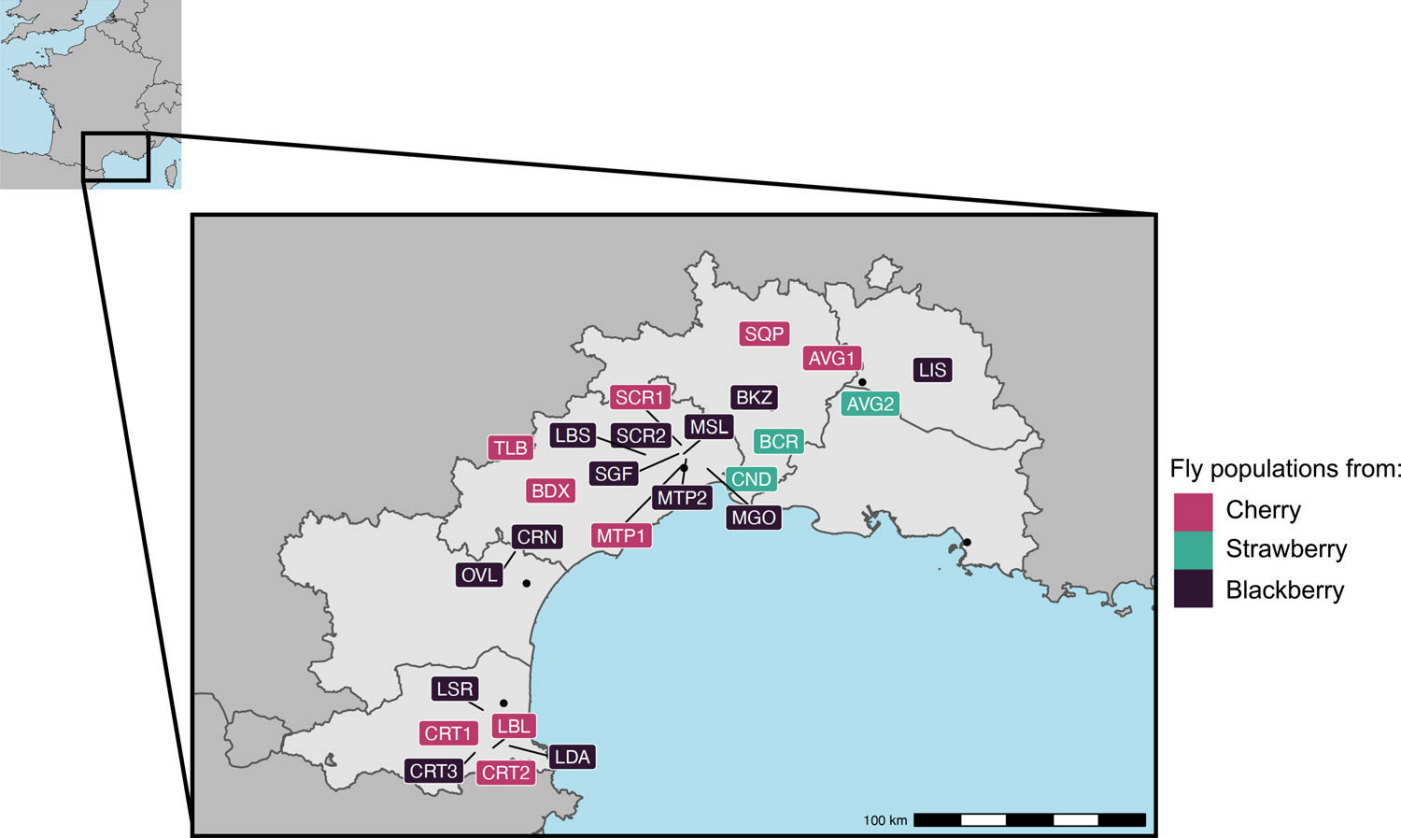
Geographic locations of the 25 sample sites where collected fruits (cherry, strawberry or blackberry) yielded enough *Drosophila suzukii* adults in the lab to be included in this study. See *Materials and Methods* and Table S6 for details.

Between May 2018 and October 2018, we collected fruits from a total of 47 sites in the South of France (Fig. 2; Table S6). The sampled sites included 20, 12, and 15 sites for cherry, strawberry, and blackberry, respectively. For each host plant, fruits were collected toward the end of the production season of the plant to maximize the number of generations over which flies could adapt to their hosts. Recent Capture‐Mark‐Recapture studies show that dispersal abilities of *D. suzukii* are generally low relative to the distance between our sampling sites (Tait et al., 2018, 2020; Vacas et al., 2019). Populations hence likely evolved on the sampled host for several generations. In addition, if we happened to sample offspring of recent immigrants, that would merely weaken our ability to detect patterns of local adaptation, and thus any such bias would be in a conservative direction. Field‐collected fruits of each population were brought back to the laboratory and kept at 21°C, 65% relative humidity, and an 18:6 (L:D) hour cycle in large plastic cages (volume ∼90 L) until adult *D. suzukii* flies emerged. This sampling scheme was used consistently throughout the entire experiment. Only the sites from which more than 150 individuals emerged were included in the experiments, leaving a total of 25 sites out of the original 47 (nine cherry, three strawberry, and 13 blackberry sites).

### RECIPROCAL COMMON ENVIRONMENT EXPERIMENT

**Figure 3:**
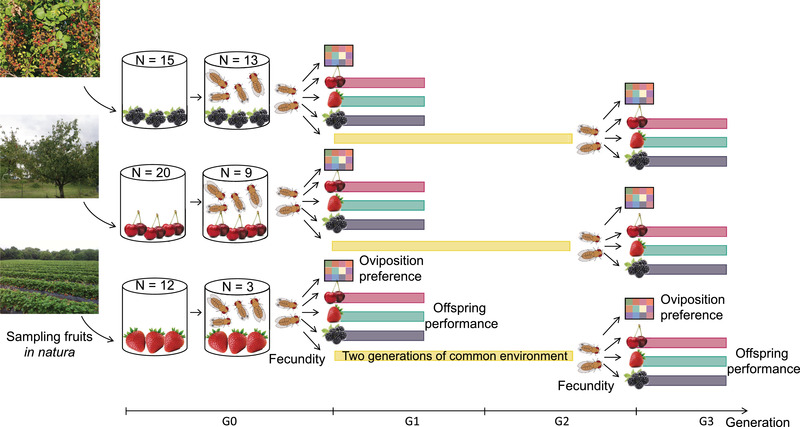
Graphical representation of the experimental design. A standard laboratory fly food medium instead of a fruit puree medium was used for the two generations in a common environment. To test for potential temporal variability in experimental conditions, a control inbred *Drosophila suzukii* line (WT3) was assayed together with each studied population (see main text for details).

We measured female preference, fecundity, and offspring performance of field‐collected flies and lab‐reared flies on purees made from original fruits (Fig. 3).

To maintain a constant quality of all fruits throughout the experiment (experimental measurements took place over several months), we prepared media using frozen purees of each of the three fruits rather than whole fruits. Batches of media were made with 600 mL of each fruit puree, supplemented with 400 mL of sterile deionized water, 1% (wt/vol) of agar, 6% of inactive malted brewer's yeast, and 1.5% of yeast extract. We then added an antimicrobial solution, which consisted of 6 mL of 99% propionic acid, 10 mL of 96% ethanol, and 0.1% of methylparaben sodium salt. For the common environment, we used a standard laboratory fly food (i.e., “German food”; Backhaus et al., 1984) including 1000 mL of sterile deionized water, 1% (wt/vol) of agar, 6% of dextrose, 3% sucrose, 8% of inactive malted brewer's yeast, 2% of yeast extract, 2% of peptone, 0.05% of MgSO_4_, 0.05% of CaCl_2_, and 16 mL antimicrobial solution (6 mL of 99% propionic acid, 10 mL 96% of ethanol, and 0.1% of methylparaben sodium salt). See Olazcuaga et al. (2019) for product references.

We collected adult flies from the field‐collected fruits after the peak of emergence (usually within 4 days after the first adults emerged). We kept only *D. suzukii* individuals and discarded other *Drosophila* species (Hauser 2011). We placed adults (hereafter referred to as “G0”) in cages with organic commercial fruits corresponding to the fruits from which the population originated and allowed them to mature for 7 days (the absence of prior *D. suzukii* infestation of these fruits was assessed by rigorous visual inspection). We performed reciprocal common environment experiments in the laboratory using (*i*) 7‐day‐old (±2 days) G0 adults that emerged from field‐collected fruits and (*ii*) 7‐day‐old (±1 day) G2 adults obtained after two generations of maintenance in a common environment (i.e., “German food”; Backhaus et al., 1984). Unlike an experiment where all populations would grow under controlled conditions in all test environments and then be measured in each test environment (i.e., a “two‐way” experimental design), the two‐generation experimental design we used enabled us to detect potential adaptive phenotypic plasticity in responses to environmental cues that are independent of the fruit itself (e.g., the photoperiod, the temperature, etc.). Using a two‐way experimental design by rearing laboratory flies on different fruits for two generations under laboratory conditions could have decreased our power to detect adaptive plasticity. For each population, we estimated female preference and offspring performance in artificial fruit media (hereafter test fruit) composed of either the fruit from which the population was sampled (hereafter original fruit) or two other alternative fruits. We measured oviposition preference and fecundity on each test fruit (see below) in G0 and G2, as proxies for female preference, and we measured egg‐to‐adult survival in G1 and G3, as a proxy for offspring performance.

Oviposition preference was measured as the number of eggs laid on different fruits by 20 females in 24 hours in an arena that contained the media of the three above mentioned host fruits plus nine other test fruit media (apricot, blackcurrant, cranberry, fig, grape, kiwi, raspberry, rose hips, and tomato) distributed randomly into 12 compartments (see fig. S2 in Olazcuaga et al. 2019). Hence, these arenas contained a wide selection of *D. suzukii* hosts that are present in the region from which wild populations were collected, expanding the inference space in which we measure oviposition preference. The detection of the oviposition preference for a fruit in these arenas is conservative, because the preference in a choice environment of 12 fruits would likely be present in a choice environment containing only three of these fruits. In addition, this experimental design has been used in other studies (e.g., Olazcuaga et al. 2019), and using this same design facilitates comparison of the oviposition preference of these wild populations with other wild or experimental populations. We also used a no‐choice assay to measure the oviposition response of females to individual fruits (hereafter fecundity), as the number of eggs laid when placing 20 adults for 24 hours in a vial with one of the three fruits. Eggs were counted under dissecting microscopes. For G0, we used on average 7.8 arenas per population to estimate oviposition preference and an average of 7.5 vials per population for the other traits. For G2, we used 7.9 arenas and 8.0 vials, respectively.

Offspring performance was measured on each fruit as egg‐to‐adult survival. We quantified egg‐to‐adult survival as the proportion of eggs in the no‐choice assay that resulted in the emergence of adults 16 days after oviposition (more than 95% of individuals emerge within 16 days on cherry, strawberry, or blackberry media; fig. 4 in Olazcuaga et al. 2019). It is worth noting that because *D. suzukii* oviposits directly into the media, removing eggs to control density was too time consuming given the size of the experiment. Furthermore, manipulation of the eggs reduces survival (Schou 2013). We therefore accounted for egg density afterward during statistical analysis (see STATISTICAL METHODS) rather than during the experiment. Egg density did not vary consistently across test or original fruits, and thus accounting for them during statistical analysis should not lead to bias. More precisely, 77.7% of the variance in the egg density was explained by the variation across tubes, whereas only 0.6% of the variance was explained by variation among test fruits and 21.7% by variation among populations.

Our field sampling design followed the seasonality of each fruit. As a result, reciprocal common environment experiments of each population were performed at different dates. To test for potential temporal variability in experimental conditions, we used an inbred *D. suzukii* line (*WT3*; Chiu et al. 2013) as a control. Together with each wild population, we measured oviposition preference, fecundity, and offspring performance in this control population. Analyses of each of the three traits of interest showed that temporal variation was of the same order of magnitude as the variation within a given date (Appendix S1).

### STATISTICAL METHODS

The analyses of oviposition preference and fecundity (log‐transformed number of eggs; Miller 1997) and offspring performance (arc‐sine‐transformed egg‐to‐adult survival; McDonald 2014) were performed using R statistical software (R Core Team 2014). For offspring performance, 51 vials had more adults than the number of eggs counted (50 of 541 vials for G0 and one of 602 for G2, with an average egg‐to‐adult survival observed of 2.44) due to a difficulty to count the eggs in the vials with very few eggs (average number of eggs of these 51 vials of 3.65). We assumed an egg‐to‐adult survival of one in the analyses of the offspring performance for these vials. Doing so is not expected to introduce a bias in the data analysis because such vials were equally represented in all treatments (17, 16, and 18 observations in cherry, strawberry, and cranberry vials, respectively). In agreement with this, processing our egg‐to‐adult survival statistical analyses provided the same qualitative results when these 51 observations were removed (results not shown).

#### Testing for trait differences between original and alternative fruits

For each trait and each generation separately, we tested whether populations had a higher preference for or performance in their original fruit than in alternative fruits. First, to visualize the patterns, we plotted the average trait in the medium corresponding to alternative fruits against the same average trait in the medium corresponding to the original fruit for each population. In addition, we computed the weighted correlation coefficient between the average trait in alternative fruits and their average in the original fruits, using the total number of vials for each population as weight, and estimated its 95% confidence interval using the *sjstats* package (Lüdecke, 2018).

Second, we tested for differences in oviposition preference, fecundity, and offspring performance of populations between original and alternative fruits using the SA method (where S stands for sympatric and A stands for allopatric) detailed in Blanquart et al. (2013). Unlike the “home‐away” and “local‐foreign” methods (Kawecki and Ebert 2004), the statistical power of the SA method does not decline with increasing sample size (number of populations; Blanquart et al. 2013). Most notably, the SA method controls for potential variation in overall quality of the test environments, a feature previously reported for hosts of *D. suzukii* (Bing et al. 2018; Olazcuaga et al. 2019). Additionally, the SA method controls for confounding factors due to quality variations among populations such as differences in inbreeding depression (Keller 2002) or differences in the prevalence of parasites or endosymbionts transmitted across generations (Fry et al. 2004; Merçot and Charlat 2004). Finally, using an ANOVA model rather than a Linear Mixed Model decreases the rate of false‐positive detection of local adaptation (appendix E in Blanquart et al. 2013).

The SA method we used is detailed in Appendix S2. Briefly, it consists of an ANOVA‐based approach to fit a model that includes population (accounting for both intrinsic genetic differences and environmental effects induced by differences among original fruits in G0/G1), test fruits (accounting for differences in dietary quality among test fruits), and an interaction between original fruits and test fruits. The model also includes a factor, called *SA*, which indicates whether the test fruit is the original (sympatric) fruit or an alternative (allopatric) fruit. The significance of the *SA* effect is tested using an *F‐*test where the ratio evaluates the variation due to *SA* over the total variation minus that due to habitat and population quality (Blanquart et al. 2013, appendix D). Positive values of *SA* indicate a pattern of local adaptation, whereas negative values indicate local maladaptation.

#### A new method to detect and quantify local adaptation and adaptive phenotypic plasticity simultaneously

To evaluate the relative contributions to preference and performance of local adaptation and adaptive phenotypic plasticity, it was necessary to analyze the data across the G0/G1 and G2/G3 generations. When measured on adults that emerged from field‐collected fruits (G0/G1), phenotypic differences among populations can be driven both by genetic differences among populations (potentially including local adaptation) and by plastic responses to the natural environment (potentially including adaptive phenotypic plasticity). When measured on adults after two generations of common environment (G2/G3), phenotypic differences among populations are likely to have a genetic basis, as potential maternal and grand‐maternal environmental effects are standardized across populations. Although transgenerational plasticity and other nongenetic factors such as vertically transmitted symbionts can still be present, we use the term “genetic effects” hereafter for the sake of brevity. To evaluate the relative contributions to preference and performance of local adaptation and adaptive phenotypic plasticity, we developed new approaches to visualize and test for genetic and plastic effects. Briefly, by using a custom model that compares the results of the two trials, the G0/G1 generation and the G2/G3 generation, we estimated the variance components and effects that could be attributed to local adaptation and adaptive phenotypic plasticity.

To visually illustrate genetic and plastic effects in preference, we estimated the mean of genetic effects based on preference data from G2 and the mean of plastic effects as the difference in preference between G0 and G2, after controlling for other sources of variation, including variation in quality among test fruits and among populations, as well as variation among arenas for oviposition preference or among vials with different egg densities for offspring performance (Appendix S3). Similarly, for performance, we estimated the mean genetic effects from G3, and the mean of plastic effects as the difference between G1 and G3.

To statistically test for genetic and plastic effects, we modified the SA method detailed in Blanquart et al. (2013) to test whether oviposition preference, fecundity, and offspring performance of populations were on average different in the original fruits and in alternative fruits and whether this effect was either genetic and present in both generations or was plastic and present in the first generation (G0 or G1), but not the second generation (G2 or G3). To this end, we derived two different test statistics for genetic and plastic effects respectively (*F_genetic_* and *F_plastic_*; see Appendix S4 for details). We performed computer simulations to assess the general properties of our method. We found that both genetic and plastic effects can be reliably detected using our method under both balanced and unbalanced experimental designs, with a false positive rate for the detection of genetic and plastic effects <3% (Fig. S5; Tables S3–S5). We also found that for unbalanced designs (as in the present study) using an ANOVA model rather than a Linear Mixed Models substantially increased the power of detecting local adaptation and adaptive phenotypic plasticity (Appendix S4).

For oviposition preference, we fitted the following ANOVA model on the log‐number of eggs, *y_ijklm_*:

(1) $$\begin{array}{ccc} y_{\mathrm{ijklm}} & = & \mathrm{popu}\mathrm{latio}n_{G2\_i}+\mathrm{test}\_\mathrm{frui}t_{G2\_j}\\ & & +\mathrm{test}\_\mathrm{fruit}\times \mathrm{orig}\mathrm{inal}\_\mathrm{frui}t_{G0G2\_\mathrm{jk}}\\ & & +\;I_{l}\Delta \mathrm{popu}\mathrm{latio}n_{G0\_i}+I_{l}\Delta \mathrm{test}\_\mathrm{frui}t_{G0\_j}\\ & & +I_{l}\Delta \mathrm{test}\_\mathrm{fruit}\times \mathrm{orig}\mathrm{inal}\_\mathrm{frui}t_{G0\_\mathrm{jk}}\\ & & +\;\mathrm{SAge}\mathrm{neti}c_{G0G2\_\mathrm{jk}}+I_{l}\mathrm{SApl}\mathrm{asti}c_{G0\_\mathrm{jk}}+\mathrm{aren}a_{m}+\varepsilon_{\mathrm{ijklm}}, \end{array}$$

where *I_l_* is an indicator variable with *I_l_* = 1 when generation *l* is G0 and *I_l_* = 0 otherwise, and fixed effects included the effect of the *i*th population estimated in G2 (*population_G2_i_* with *i* = 1, …, 25) to account for differences in quality among populations, the *j*th fruit media estimated in G2 (*test_fruit_G2_j_* with *j* = 1, …, 3 for blackberry, cherry, and strawberry, respectively) to account for differences in quality among test fruits, the interaction between the *j*th test fruit and the *k*th original fruit that is observed in both G0 and G2 (*test_fruit*×*original_fruit_G0G2_jk_*), and the plastic difference of these effects in G0 relative to the same effects in G2 (*∆population_G0_i_*, *∆test_fruit_G0_j_*, and *∆test_fruit*×*original_fruit_G0_jk_*, respectively). Fixed effects also included two SA effects, which indicated whether the test fruit was the original, sympatric fruit or was an alternative, allopatric fruit in either G0 or G2 (*SAgenetic_G0G2_jk_*) and in G0 (*SAplastic_G0_jk_*). Hence, *SAgenetic_G0G2_jk_* measures local adaptation in both G0 and G2 and *SAplastic_G0_jk_* measures adaptive phenotypic plasticity in G0 (i.e., field‐collected flies). The model also included the fixed effect of the *m*th arena in G0 and G2 (*arena_m_* with *m* = 1, …, 179 in G0 and *m* = 180, …, 369 in G2) to account for differences among arenas and a random error to account for the variation among observations from the same arena (*ε_ijklm_*, normally distributed with a mean of zero and variance *σ* ^2^ *_res_*). As with the original SA method, this new method detects if there is a significant difference when the test fruit is the original fruit versus an alternative fruit. A significant difference in the associated *F*‐test with a positive estimate of *SAgenetic_G0G2_* and *SAplastic_G0_* provides support in favor of local adaptation and adaptive phenotypic plasticity, respectively. We either analyzed the entire dataset (12 fruits) or focused on a smaller dataset with the three fruits of interest (cherry, strawberry, and blackberry). Results were similar in both analyses, so we only present the results with only the three fruits of interest.

For fecundity, we fitted the same model but without the arena effect. For offspring performance, we fitted the same model with G1 and G3. Offspring performance increases with egg density and levels off at high egg densities (Fig. S12). To account for egg density in the statistical analysis of offspring performance, we replaced the arena effect by the log‐transformed initial number of eggs (*eggs_m_*).

To estimate the level of local adaptation, we designed a new variable *φ_genetic_* based on the proportion of variance of the interaction *test_fruit*×*original_fruit_G0G2_jk_* explained by *SAgenetic_G0G2_jk_*:

(2) $$\varphi_{\mathrm{gene}\mathrm{tic}}=\frac{\mathrm{Mean}\mathrm{Squar}e_{\mathrm{SAge}\mathrm{neti}c_{G0G2\_\mathrm{jk}=0}}-\mathrm{Mean}\mathrm{Squar}e_{\mathrm{SAge}\mathrm{neti}c_{G0G2\_\mathrm{jk}\neq 0}}}{\mathrm{Mean}\mathrm{Squar}e_{\mathrm{SAge}\mathrm{neti}c_{G0G2\_\mathrm{jk}=0}}}.$$

Similarly, the level of adaptive phenotypic plasticity *φ_plastic_* was estimated based on the proportion of variance of the interaction *test_fruit*×*original_fruit_G0_jk_* explained by *SAplastic_G0_jk_*:

(3) $$\varphi_{\mathrm{plas}\mathrm{tic}}=\frac{\mathrm{Mean}\mathrm{Squar}e_{\mathrm{SApl}\mathrm{asti}c_{G0\_\mathrm{jk}=0}}-\mathrm{Mean}\mathrm{Squar}e_{\mathrm{SApl}\mathrm{asti}c_{G0\_\mathrm{jk}\neq 0}}}{\mathrm{Mean}\mathrm{Squar}e_{\mathrm{SApl}\mathrm{asti}c_{G0\_\mathrm{jk}=0}}}.$$

Because we did not detect any significant adaptive phenotypic plasticity (see results), we retrospectively performed a simulation‐based analysis to estimate our power to detect the presence of adaptive phenotypic plasticity with our experimental design (Appendix S5).

#### Testing for correlations between generation and between fitness related traits

We tested for two sets of correlations. First, we evaluated whether there is a correlation in oviposition preference, fecundity, or offspring performance of populations between generations (Appendix S6). Second, we examined whether the evolution of preference and performance traits is independent or rather if they coevolve. Specifically, we tested for a significant correlation between oviposition preference and offspring performance between G0 and G1, and between G2 and G3 (Appendix S7). In each of these two cases, we estimated correlations using the total number of vials for each population as a weight and estimating its 95% confidence interval using the *sjstats* package (Lüdecke, 2018).

## Results

### TEST FOR TRAIT DIFFERENCES BETWEEN ORIGINAL AND ALTERNATIVE FRUITS

**Figure 4:**
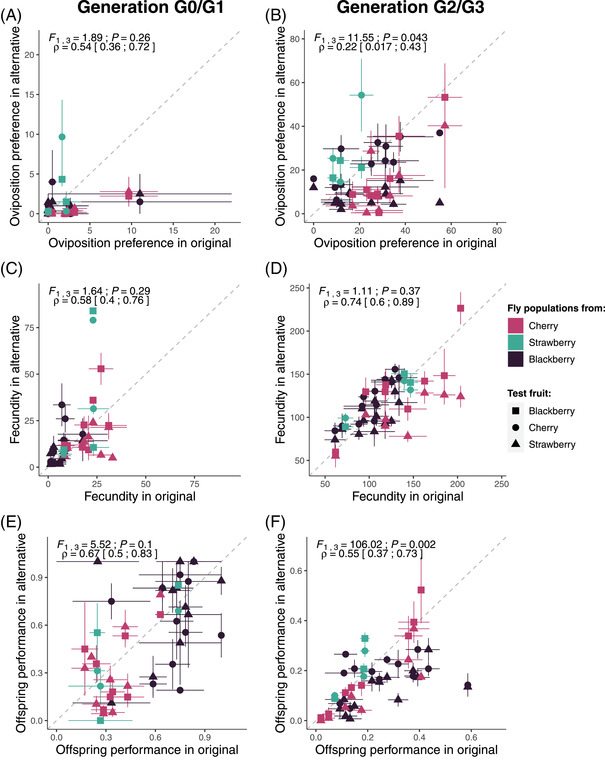
Relationship between original and alternative fruits for (A, B) oviposition preference (number of eggs laid by 20 females in 24 hours in the original or alternative fruit media, in a choice environment), (C, D) fecundity (number of eggs laid by 20 females in 24 hours in the original or alternative fruit media, in a no‐choice environment), and (E, F) offspring performance (egg‐to‐adult survival) in generations G0 or G1 (left panels) and G2 or G3 (right panels). The term “original” in the *x*‐axis legend indicates that the trait is measured in the same fruit from which populations were collected, whereas the term “alternative” in the *y*‐axis legend indicates measurements on other fruits. The mean of each population in each test fruit is represented by a symbol whose shape depends on the test fruit (fruit medium) and color depends upon the fruit from which the population was collected in natura. For each population, the two means measured in the two alternative fruits are associated with the same and unique mean in the original fruit. Local adaptation corresponds to the cases where the means measured in the two alternative fruits are both located below the line of symmetry, due to a higher mean trait value in the original fruit. Error bars represent the standard error of mean estimates. Results (*F*‐statistics and *P*‐values) from the SA method detailed in Blanquart et al. (2013), as well as the weighted Pearson's correlation coefficient (*ρ*) with their 95% confidence interval, are given above each panel.

Across the three traits of interest (oviposition preference, fecundity, and offspring performance) and for each generation separately, populations with higher trait values in their original fruit generally had higher trait values in alternative fruits (Fig. 4). Indeed, the correlation coefficients between traits in original and alternative fruits were positive and significantly different from zero for all the traits and generations (Fig. 4). These positive correlations indicate variation in intrinsic quality among populations within and among fruits and, most importantly, emphasize the importance of statistically accounting for this variation when testing for local adaptation and adaptive phenotypic plasticity (Appendix S2). For oviposition preference, these correlations among populations were higher in G0 than in G2 for oviposition preference (*ρ*_G0_ = 0.54 and *ρ*_G2_ = 0.22; Fig. 4A vs. 4B). In contrast, for fecundity, the correlations among populations were weaker, with more variation observed in G0 than in G2 (*ρ*_G0_ = 0.58 and *ρ*_G2_ = 0.74; Fig. 4C vs. 4D), suggesting greater phenotypic plasticity in this trait in flies collected from the field. Finally, these correlations among populations were higher in G1 than in G3 for offspring performance (*ρ*_G1_ = 0.67 and *ρ*_G3_ = 0.55; Fig. 4E vs. 4D).

In G0 and G1, the values of the three traits of interest did not significantly differ between original and alternative fruits (Figs. 4A,C,E, S5A,C,E). In G2 and G3, most of the populations preferred to oviposit and had higher offspring performance in their original fruit than in alternative fruit media (i.e., most populations are under the dashed line of symmetry in Fig. 4B,F). This pattern was particularly marked for offspring performance (*F*_1,3_ = 106.02, *P* = 0.002; Fig. S5F), but more tenuous for oviposition preference (*F*_1,3_ = 11.55, *P* = 0.043; Fig. S5B). In contrast, no evidence for higher fecundity on the original fruit than on alternative fruit media was found (*F*_1,3_ = 1.11, *P* = 0.37; Figs. 4D, S5D).

### RELATIVE IMPORTANCE OF LOCAL ADAPTATION AND ADAPTIVE PHENOTYPIC PLASTICITY

**Figure 5:**
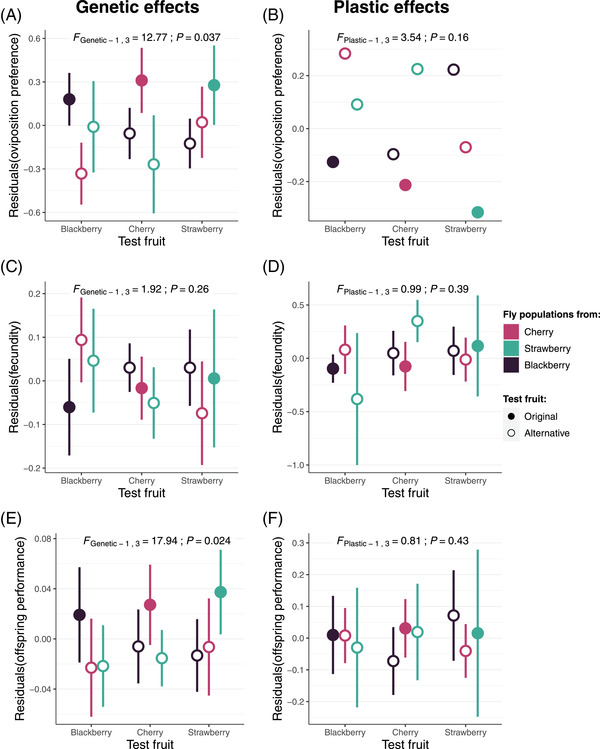
Genetic (left panels) and plastic (right panels) effects across *Drosophila suzukii* natural populations for (A, B) oviposition preference, (C, D) fecundity, and (E, F) offspring performance for each combination of original fruit and test fruit. Genetic effects (i.e., testing for local adaptation) correspond to each trait value in G2 or G3, whereas plastic effects (i.e., testing for adaptive phenotypic plasticity) correspond to the differences between each trait value in G0 or G1 and their respective trait value in G2 or G3. For both genetic and plastic effects, we ran models to account for other sources of variation, including variation in quality among test fruits and among populations, as well as variation among arenas for oviposition preference or among vials with different egg densities for offspring performance. The residuals from these models are represented on the *y*‐axis. Results (*F*‐statistics and *P*‐values) for the test of adaptive phenotypic plasticity or local adaptation are given above each panel. Error bars represent 95% confidence intervals. For plastic effects of oviposition preference, 95% confidence intervals could not be computed due to a higher variance in G2 than in G0.

When analyzing G0 and G2 data together (Fig. 5), our new statistical method showed that the higher preference for their original fruit media had a significant genetic origin (*P* = 0.037 with a positive estimate of *SAgenetic_G0G2_*; Table 1). Overall, local adaptation (*SAgenetic_G0G2_*) explained 74.64% of the variance of the genetic interaction between the test and original fruits in G0 and G2. Although phenotypic plasticity was in the maladaptive direction (negative estimate of *SAplastic_G0_*; Table 1), it was not significantly different from zero (*P* = 0.16).

**Table 1:** Estimates for *SAgenetic* and *SAplastic* for oviposition preference, fecundity, and offspring performance

	Oviposition Preference	Fecundity	Offspring Performance
*φ_genetic_*	74.64%	18.67%	80.90%
*φ_plastic_*	38.81%	−0.27%	−5.01%
*F_genetic_*	12.77	1.92	17.94
*P*‐value	0.037	0.26	0.024
*SAgenetic*	0.384	−0.058	0.042
SE*_SAgenetic_*	0.08	0.063	0.028
*F_plastic_*	3.54	0.99	0.81
*P*‐value	0.16	0.39	0.43
*SAplastic*	−0.266	−0.132	0.043
SE*_SAplastic_*	0.116	0.099	0.044

*Note*: The *SAgenetic* and *SAplastic* estimates are presented with their standard error (SE*_SAgenetic_* and SE*_SAplastic_*, respectively). The *F_genetic_* and *F_plastic_* on *SAgenetic* and *SAplastic* and corresponding *P*‐values are also indicated.

For fecundity, the absence of pattern in G0 and G2 when analyzed independently (Fig. S5) was confirmed, as no significant genetic or plastic effects were detected when analyzing G0 and G2 data together (Fig. 5C,D; *P* = 0.26 and *P* = 0.39, respectively; Table 1).

The higher performance of the flies on their original fruit media (Fig. 5F) had a significant genetic origin (*P* = 0.024, with a positive estimate of *SAgenetic_G1G3_*; Table 1). Overall, local adaptation (*SAgenetic_G1G3_*) explained 80.9% of the variance of the genetic interaction between the test and original fruits in both G1 and G3. Although phenotypic plasticity was in the adaptive direction for this trait (positive estimate of *SAplastic_G1_*; Table 1), it was not significantly different from zero (*P* = 0.43).

### TESTING FOR CORRELATIONS BETWEEN FEMALE PREFERENCE AND OFFSPRING PERFORMANCE

We tested for a correlation between oviposition preference and offspring performance, after controlling for other sources of variation, including variation in quality among test fruits and among populations, as well as variation among arenas for oviposition preference or among vials with different egg densities for offspring performance (see Appendix S7 for details). We found that oviposition preference in G0 was positively correlated with offspring performance in G1, and oviposition preference in G2 was positively correlated with offspring performance in G3 (Fig. S9). The correlation coefficient was significantly different from zero for the G2/G3 generation test, whereas the 95% confidence intervals of the correlation coefficient included zero for the G0/G1 generation test.

## Discussion

We used natural populations of *D. suzukii* to evaluate the roles of local adaptation and adaptive phenotypic plasticity in driving fitness adjustment to changes in the environment occurring over an intermediate pace of three to four generations. We focused on three key traits: oviposition preference, fecundity, and offspring performance. We found local adaptation for oviposition preference and offspring performance, but not for fecundity. Females preferred their original fruits, and their offspring performed better on those fruits. We did not find any signature of adaptive phenotypic plasticity for any of the three traits. If adaptive phenotypic plasticity plays a role in the fit to the environment, that role is small enough to be masked by the effects of non‐adaptive plasticity and local adaptation.

### STRONG VARIATION IN QUALITY AMONG POPULATIONS IN ALL TESTED FRUITS

For each of the three traits, *D. suzukii* populations displayed consistent correlations between the original and test fruits. Some populations had the stronger preference or performance across tested fruits, highlighting the high variation in intrinsic quality among populations. *Drosophila suzukii* population quality may be related to various ecological and historical factors, as well as their genetic background, with some populations having, for instance, low genetic load and others having higher genetic load. The level of inbreeding depression, which depends on the level of genetic load, is indeed known to vary among wild populations and influences life‐history traits (Keller and Waller 2002).

### THE HIGHER PREFERENCE AND PERFORMANCE IN ORIGINAL FRUIT MEDIA ARE DRIVEN BY LOCAL ADAPTATION RATHER THAN ADAPTIVE PHENOTYPIC PLASTICITY

We found that the stronger oviposition preference for and higher performance in the original fruit than in alternative fruits were due to genetic and not plastic responses. Our research is relevant to current understanding of how the tempo of environmental change over time and space (i.e., environmental grain) shapes the way organisms improve the match between their phenotypic traits and their local environment. Based on the period of availability of each host fruit, average temperatures in the studied areas, and the generation time estimates in the laboratory (Poyet et al. 2015; Olazcuaga et al. 2019), our results indicate that local adaptation (and not just adaptation) can evolve in less than four generations in large diverse natural populations of *D. suzukii*. This indicates that the evolutionary process of local adaptation, including lower relative fitness in alternative fruits, can be more rapid than traditionally thought (e.g., 10 generations in natural populations of *D. melanogaster*; Bergland et al. 2014). In agreement with this, rapid adaptation over only a few generations has been recently documented in populations of *D. melanogaster* evolving in seminatural conditions (Rudman et al. 2022) and natural conditions (Machado et al. 2021).

It is likely that at the end of the season of a given fruit, the *D. suzukii* populations established on this fruit will switch to another fruit resource located in the neighborhood because diapause has never been observed for this species between spring and the end of summer (Walsh et al. 2011). Supporting this interpretation is the fact that genetic differentiation among populations sampled on different fruits is weak (unpubl. data). Therefore, because populations have to switch to a new fruit regularly due to the seasonality of fruits, the rapid and dynamic process of local adaptation in *D. suzukii* seems to be transient. Transient and rapid local adaptation has also been found in experimental populations of another crop pest species *Tetranychus urticae* maintained in the lab in a temporally heterogeneous environment with two host plants (cucumber or pepper) in succession (Bisschop et al. 2019). At the genome level, the seasonality of host fruits might result in balancing selection at adaptive loci and thus help to maintain high standing genetic variation in natural *D. suzukii* populations at and around those loci (Gloss et al. 2013).

The rapid genetic adaptation we observed could be favored by *D. suzukii* populations having a large effective size and by strong and divergent selective pressure exerted by seasonal fruits. The population size of *D. suzukii* in each of the studied sites is likely to be large (on the order of 10^4^ adults according to estimates from American cherry orchards; Tochen et al. 2014; Wiman et al. 2014) as suggested by the high levels of genetic diversity observed in *D. suzukii* populations sampled in the South of France (Fraimout et al. 2017; Olazcuaga et al. 2020). Differences in chemical composition among fruits likely result in strong pressures on traits such as offspring performance, as evidenced by a recent experimental evolution study (Olazcuaga et al. 2021).

We did not detect a correlation between trait values measured in generations G0 and G2 for preference traits or in G1 and G3 for performance for a given population. This finding can be explained by the presence of a substantial amount of non‐adaptive plasticity in G0 (Fig. 1C), which masked the genetic effects responsible for phenotypic patterns of local adaptation. In addition, we did not detect adaptive phenotypic plasticity, although we had enough statistical power to detect an effect of the same magnitude as local adaptation. The failure to detect adaptive phenotypic plasticity may seem surprising at first sight because *Drosophila* species, including *D. suzukii*, usually excel at using cues to optimize their oviposition behavior (Jaenike 1983; Papaj and Rausher 1983; Little et al. 2020). The absence of evidence for adaptive phenotypic plasticity suggests that in the case of *D. suzukii* the changes in plant hosts are not frequent enough for plasticity to be strongly beneficial. In addition, the flies were sampled in an agricultural region in France that tends to exhibit monocultures of crops rather than polycultures, which might also contribute to a lack of plasticity, as different crop fruits are rarely available at the same time within the same area.

### RELATIONSHIPS BETWEEN FEMALE OVIPOSITION PREFERENCE AND OFFSPRING PERFORMANCE

We found that oviposition preference in generation G2 was positively and significantly correlated with offspring performance in generation G3. Previous studies that tested for this correlation in *D. suzukii* have shown mixed results (Poyet et al. 2015; Olazcuaga et al. 2019; Shu et al. 2022). Our results, based on natural populations of *D. suzukii*, suggest that selection pressures on oviposition preference might drive adaptation to host fruits and could speed up local adaptation. This is consistent with previous theoretical and experimental studies showing that selective pressures on oviposition preference could feedback positively on the evolution of performance for local hosts (Whitlock 1996; Berlocher and Feder 2002; Via and Hawthorne 2002). In particular, if preference and performance traits are genetically linked, strong selection pressures on oviposition preference, at least for the three fruits studied here, could lead to the evolution of performance traits (Wood et al. 1999; Berner and Thibert‐Plante 2015).

### LIMITS OF OUR EXPERIMENTAL APPROACH

Our experiments do not overcome the common drawback of trying to infer the fitness of natural populations using experiments in the laboratory rather than in natural conditions (Reznick and Ghalambor 2005). First, the small scale of our setup for studying choice among multiple fruits deviates considerably from the conditions and geographic scales found in natural landscapes and may therefore provide an incomplete picture of the adaptive processes regarding oviposition preference in *D. suzukii*. Second, our experiments did not consider yeasts and bacteria that occur in fruits in the field, and which have been shown to modify the foraging behavior (Shu et al. 2021) as well as the oviposition preference (Bellutti et al. 2018) of *D. suzukii* females. Yeasts and bacteria can also affect performance, as microbes represent an important source of protein for the egg‐to‐adult survival of *D. suzukii* (Lewis and Hamby 2019; Bing et al. 2021). Therefore, if the microbial community differs among cherry, strawberry, and blackberry, our results could be at least partly explained by the nutritional effect of a fly microbiome transmitted over generations rather than a *D. suzukii‐*based genetic effect. Experiments in *D. melanogaster* have shown that its bacterial community can be transmitted over successive generations in the laboratory (Téfit et al. 2018) and that both *Acetobacter* and *Lactobacilli* can grow in media with concentration of antimicrobials similar to the one in our media (Obadia et al. 2018). Using axenic individuals could help testing for the role of the microbiome in the patterns we detected, although the absence of microbiota itself could have an important impact on both preference and performance.

## Conclusion

We studied oviposition preference, fecundity, and offspring performance in *D. suzukii* to evaluate the relative influence of genetic and plastic effects on the match between phenotypic traits and the local environment, here the host fruit. We found a pattern of local adaptation for oviposition preference and offspring performance, but not for fecundity. We found no evidence of adaptive phenotypic plasticity for all studied traits. Our study hence demonstrates that spatially and temporally variable selection does not prevent the rapid evolution of local adaptation in natural populations over a short number of generations. This dynamic process appears to be transient as populations rapidly adapt to new host fruits as they become available throughout the year. In addition to testing for the potential role of the microbiome in the pattern we detected, a stimulating follow‐up to our study would be to identify the physiological pathways under selection using genome‐environment association study based on populations sampled from different host plants. Such a combination of statistical, molecular, and quantitative approaches would provide useful insights into the genomic and phenotypic responses to divergent selection among host fruits in phytophagous generalist insects. Finally, our new statistical method will likely contribute to the quantification of the relative contribution of local adaptation and adaptive phenotypic plasticity in any other organisms experiencing spatially or temporally variable selection.

## AUTHOR CONTRIBUTIONS

LO, JF, MG, RAH, NOR, and AE conceptualized the idea of the study and acquired funding. LO performed statistical analyses and wrote the manuscript with inputs from JF, MG, RAH, NOR, and AE. LO, CD, AL, J‐LI, RV, RG, CS, and AE performed data acquisition and field sampling.

## DATA ARCHIVING

The data and R scripts for our analyses are available at: https://github.com/nrode/NatPop2021 and in the Dryad Repository: https://doi.org/10.5061/dryad.2z34tmpqh.

## CONFLICT OF INTEREST

The authors declare no conflict of interest.

## Supporting information

Appendix S1: Variation in environmental effects throughout the experiment.
Table S1. Variance analysis for the three traits of interest i.e., oviposition preference, fecundity and offspring performance.
Figure S1. (A) Oviposition preference (number of eggs laid by 20 females in 24 hours in a choice environment), (B) fecundity (number of eggs laid by 20 females in 24 hours in a no‐choice environment), and (C) offspring performance (egg‐to‐adult survival) measured in the *D. suzukii* inbred line WT3 for each date of reciprocal transplant experiments.
Figure S2. Residuals of (A) Oviposition preference, (B) fecundity, and (C) offspring performance, measured in the *D. suzukii* inbred line WT3 for each date of reciprocal transplant experiments.
Appendix S2: Testing for a pattern of local adaptation by considering the variation in intrinsic quality among test fruits and among populations.
Figure S3. (A‐B) Oviposition preference (number of eggs laid by 20 females in 24 hours on the original or alternative fruit, in a choice environment), (C‐D) fecundity (number of eggs laid by 20 females in 24 hours on the original or alternative fruit, in a no‐choice environment), and (E‐F) offspring performance (egg‐to‐adult survival) of *D. suzukii* natural populations for each combination of original fruit and test fruit.
Figure S4. Difference in (A‐B) oviposition preference (number of eggs laid by 20 females in 24 hours in a choice environment), (C‐D) fecundity (number of eggs laid by 20 females in 24 hours in a no‐choice environment), and (E‐F) offspring performance (egg‐to‐adult survival) between original and alternative fruits for each population in generations G0 or G1 (left panels) and G2 or G3 (right panels).
Figure S5. Residual variation after statistically controlling for intrinsic differences in environment and population quality of (A‐B) oviposition preference, (C‐D) fecundity, and (E‐F) offspring performance of *D. suzukii* natural populations for each combination of original fruit and test fruit either before (G0 and G1, left panel) or after the common environment (G2 and G3, right panel).
Figure S6. First and second axes of the Principal Component Analysis (PCA) of all populations across the three test fruits for (A‐B) oviposition preference, (C‐D) fecundity, and (E‐F) offspring performance in generations G0/G1 (left panels) and G2/G3 (right panels).
Appendix S3: Estimation of genetic and plastic effects for each combination of test fruit x original fruit.
Appendix S4: Statistical power of a new test to detect local adaptation and adaptive phenotypic plasticity based on the *F_genetic_* and *F_plastic_* tests.
Table S2. Parameters values for the different distributions.
Figure S7. Power analyses for the tests on both genetic and plastic effects for balanced and unbalanced designs for each of three data distributions: Normal, Poisson and Binomial distribution of data. Anova: *F*‐test based on an ANOVA model. LMM: *F*‐test based on a Linear Mixed Model (LMM).
Figure S8. Power analyses for the tests on both genetic and plastic effects with an arena random effect (Poisson distribution of data).
Table S3. False positive rates for the tests of *SAgenetic_G0G2_* and *SAplastic_G0_* for each of the three distributions of data.
Table S4. False positive rates for the test of *SAgenetic_G0G2_* for each of the three distributions of data.
Table S5. False positive rates for the test of *SAplastic_G0_* with each of the three distributions of data.
Appendix S5: Statistical power to detect adaptive phenotypic plasticity with our experimental setup.
Figure S9. Estimation of the power of detecting plastic effects for different *SAplastic_G0_* values under our experimental design (X1) and under larger designs (X2, X10 and X100) using simulated data.
Appendix S6: Correlation across generations for each trait.
Figure S10. Relationship between (A) oviposition preference in G0 and G2, (B) fecundity in G0 and G2, and (C) offspring performance in G1 and G3 across the three test fruits.
Appendix S7: Test of the “Mother knows best” hypothesis.
Figure S11. Correlation between oviposition preference and offspring performance (A) in generation G0/G1 and (B) in generation G2/G3.
Table S6: Key information about the 25 *D. suzukii* populations collected in the wild that were subsequently analyzed in the laboratory.
Figure S12. Effect of egg density on offspring performance for each test fruit.

Supporting information

## References

[evl3304-bib-0001] Aly, M. F. K. (2018). Laboratory host selection and development of immature *Drosophila suzukii* (Diptera: Drosophilidae) on fruits and artificial diets. J. Phytopathol. Pest Manag., 5, 129–142.

[evl3304-bib-0002] Auld, J. R. , A. A. Agrawal & R. A. Relyea . (2010). Re‐evaluating the costs and limits of adaptive phenotypic plasticity. Proc. R. Soc. Edinburgh, Sect. B: Biol. Sci., 277, 503–511. 10.1098/rspb.2009.1355 PMC2842679 19846457

[evl3304-bib-0082] Backhaus, B. , Sulkowski, E. , Schlote, F. , (1984). A semi‐synthetic, general‐purpose medium for Drosophila melanogaster. Dros Inf Serv.

[evl3304-bib-0003] Barghi, N. , J. Hermisson & C. Schlötterer . (2020). Polygenic adaptation: a unifying framework to understand positive selection. Nat Rev Genet, 21, 769–781. 32601318 10.1038/s41576-020-0250-z

[evl3304-bib-0004] Bellutti, N. , A. Gallmetzer , G. Innerebner , S. Schmidt , R. Zelger & E. H. Koschier . (2018). Dietary yeast affects preference and performance in *Drosophila suzukii* . J. Pest Sci., 91, 651–660. 10.1007/s10340-017-0932-2 PMC5847167 29568250

[evl3304-bib-0005] Bergland, A. O. , E. L. Behrman , K. R. O'Brien , P. S. Schmidt & D. A. Petrov . (2014). Genomic evidence of rapid and stable adaptive oscillations over seasonal time scales in *Drosophila* . PLoS Genet., 10, e1004775. 25375361 10.1371/journal.pgen.1004775 PMC4222749

[evl3304-bib-0006] Berlocher, S. H. & J. L. Feder . (2002). Sympatric speciation in phytophagous insects: moving beyond controversy? Annu. Rev. Entomol., 47, 773–815. 11729091 10.1146/annurev.ento.47.091201.145312

[evl3304-bib-0007] Berner, D. & X. Thibert‐Plante . (2015). How mechanisms of habitat preference evolve and promote divergence with gene flow. J. Evol. Biol., 28, 1641–1655. 26119841 10.1111/jeb.12683 PMC4573339

[evl3304-bib-0008] Bing, X. , J. Gerlach , G. Loeb & N. Buchon . (2018). Nutrient‐dependent impact of microbes on *Drosophila suzukii* development. mBio., 9, e02199–e02117. 29559576 10.1128/mBio.02199-17 PMC5874910

[evl3304-bib-0009] Bing, X.‐L. , J. Winkler , J. Gerlach , G. Loeb & N. Buchon . (2021). Identification of natural pathogens from wild *Drosophila suzukii* . Pest Manage. Sci., 77, 1594–1606. 10.1002/ps.6235 33342014

[evl3304-bib-0010] Bisschop, K. , F. Mortier , R. S. Etienne & D. Bonte . (2019). Transient local adaptation and source–sink dynamics in experimental populations experiencing spatially heterogeneous environments. Proc. R. Soc. Bio. Sci., 286, 20190738. 10.1098/rspb.2019.0738 PMC6599998 31238842

[evl3304-bib-0011] Blanquart, F. , O. Kaltz , S. L. Nuismer & S. Gandon . (2013). A practical guide to measuring local adaptation. Ecol Lett., 16, 1195–1205. 23848550 10.1111/ele.12150

[evl3304-bib-0012] Bonnet, X. , F. Brischoux , M. Briand & R. Shine . (2021). Plasticity matches phenotype to local conditions despite genetic homogeneity across 13 snake populations. Proc. R. Soc. Bio. Sci., 288, 20202916. 10.1098/rspb.2020.2916 PMC7893287 33499786

[evl3304-bib-0013] Botero, C. A. , F. J. Weissing , J. Wright & D. R. Rubenstein . (2015). Evolutionary tipping points in the capacity to adapt to environmental change. Proc. Natl. Acad. Sci., 112, 184–189. 25422451 10.1073/pnas.1408589111 PMC4291647

[evl3304-bib-0014] Burrack, H. J. , G. E. Fernandez , T. Spivey & D. A. Kraus . (2013). Variation in selection and utilization of host crops in the field and laboratory by *Drosophila suzukii* Matsumara (Diptera: Drosophilidae), an invasive frugivore. Pest Manage. Sci., 69, 1173–1180. 10.1002/ps.3489 23494939

[evl3304-bib-0015] Chiu, J. C. , X. Jiang , L. Zhao , C. A. Hamm , J. M. Cridland , P. Saelao , et al. (2013). Genome of *Drosophila suzukii*, the spotted wing drosophila. G3, 3, 2257–2271. 24142924 10.1534/g3.113.008185 PMC3852387

[evl3304-bib-0016] Davis, J. M. & J. A. Stamps . (2004). The effect of natal experience on habitat preferences. Trends Ecol. Evol., 19, 411–416. 16701298 10.1016/j.tree.2004.04.006

[evl3304-bib-0017] Denno, R.F. & Dingle, H. (Eds.). (1981). Insect life history patterns: habitat and geographic variation. Proceedings in life sciences. Springer, New York.

[evl3304-bib-0018] Dewitt, T. J. , A. Sih & D. S. Wilson . (1998). Costs and limits of phenotypic plasticity. Trends Ecol. Evol., 13, 77–81. 21238209 10.1016/s0169-5347(97)01274-3

[evl3304-bib-0019] Dury, G. J. & M. J. Wade . (2020). When mother knows best: a population genetic model of transgenerational versus intragenerational plasticity. J. Evol. Biol., 33, 127–137. 31549475 10.1111/jeb.13545 PMC7891633

[evl3304-bib-0020] Enbody, E. D. , M. E. Pettersson , C. G. Sprehn , S. Palm , H. Wickström & L. Andersson . (2021). Ecological adaptation in European eels is based on phenotypic plasticity. Proc. Natl. Acad. Sci., 118, e2022620118. 33479174 10.1073/pnas.2022620118 PMC7848574

[evl3304-bib-0021] Fraimout, A. , V. Debat , S. Fellous , R. A. Hufbauer , J. Foucaud , P. Pudlo , et al. (2017). Deciphering the routes of invasion of *Drosophila suzukii* by means of ABC random forest. Mol. Biol. Evol., 34, 980–996. 28122970 10.1093/molbev/msx050 PMC5400373

[evl3304-bib-0022] Fry, A. J. , M. R. Palmer & D. M. Rand . (2004). Variable fitness effects of *Wolbachia* infection in *Drosophila melanogaster* . Heredity, 93, 379–389. 15305172 10.1038/sj.hdy.6800514

[evl3304-bib-0023] Fry, J. D. (1996). The evolution of host specialization: are trade‐offs overrated? Am. Nat., 148, 84–107.

[evl3304-bib-0024] Gavrilets, S. & S. M. Scheiner . (1993). The genetics of phenotypic plasticity. V. Evolution of reaction norm shape. J. Evol. Biol., 6, 31–48.

[evl3304-bib-0025] Gillespie, J. (1974). The role of environmental grain in the maintenance of genetic variation. Am. Nat., 108, 831–836.

[evl3304-bib-0026] Gloss, A. D. , A. C. N. Dittrich , B. Goldman‐Huertas & N. K. Whiteman . (2013). Maintenance of genetic diversity through plant‐herbivore interactions. Curr. Opin. Plant Biol., 16, 443–450. 23834766 10.1016/j.pbi.2013.06.002 PMC4059408

[evl3304-bib-0027] Hansen, T. F. , A. J. R. Carter & C. Pélabon . (2006). On adaptive accuracy and precision in natural populations. Am. Nat., 168, 168–181. 16874627 10.1086/505768

[evl3304-bib-0028] Hauser, M. (2011). A historic account of the invasion of *Drosophila suzukii* (Matsumura) (Diptera: Drosophilidae) in the continental United States, with remarks on their identification. Pest Manage. Sci., 67, 1352–1357. 10.1002/ps.2265 21898759

[evl3304-bib-0029] Hereford, J. (2009). A quantitative survey of local adaptation and fitness trade‐offs. Am. Nat., 173, 579–588. 19272016 10.1086/597611

[evl3304-bib-0030] Jaenike, J. (1978). On optimal oviposition behavior in phytophagous insects. Theor. Popul. Biol., 14, 350–356. 751265 10.1016/0040-5809(78)90012-6

[evl3304-bib-0031] Jaenike, J. (1983). Induction of host preference in *Drosophila melanogaster* . Oecolog., 58, 320–325. 10.1007/BF00385230 28310329

[evl3304-bib-0032] Jong, G. D. (1999). Unpredictable selection in a structured population leads to local genetic differentiation in evolved reaction norms. J. Evol. Biol., 12, 839–851.

[evl3304-bib-0033] Kawecki, T. J. & D. Ebert . (2004). Conceptual issues in local adaptation. Ecol Lett., 7, 1225–1241.

[evl3304-bib-0034] Keller, L. (2002). Inbreeding effects in wild populations. Trends Ecol. Evol., 17, 230–241.

[evl3304-bib-0035] Kenis, M. , L. Tonina , R. Eschen , B. v. d. Sluis , M. Sancassani , N. Mori , et al. (2016). Non‐crop plants used as hosts by *Drosophila suzukii* in Europe. J. Pest. Sci., 89, 735–748. 10.1007/s10340-016-0755-6 PMC5318492 28275324

[evl3304-bib-0036] Lee, J. C. , D. J. Bruck , A. J. Dreves , C. Ioriatti , H. Vogt & P. Baufeld . (2011). In focus: spotted wing drosophila, *Drosophila suzukii*, across perspectives. Pest Manage. Sci., 67, 1349–1351. 10.1002/ps.2271 21990168

[evl3304-bib-0037] Levins, R. (1968). Evolution in changing environments: some theoretical explorations. Princeton Univ. Press, Princeton, NJ.

[evl3304-bib-0038] Lewis, M. T. & K. A. Hamby . (2019). Differential impacts of yeasts on feeding behavior and development in larval *Drosophila suzukii* (Diptera: Drosophilidae). Sci. Rep., 9, 1–12. 31527678 10.1038/s41598-019-48863-1 PMC6746873

[evl3304-bib-0039] Little, C. M. , T. W. Chapman & N. K. Hillier . (2020). Plasticity is key to success of *Drosophila suzukii* (Diptera: Drosophilidae) invasion. J Insect Sci., 20, 5. 10.1093/jisesa/ieaa034 PMC7230767 32417920

[evl3304-bib-0080] Lüdecke, D. (2018). Sjstats: Statistical Functions For Regression Models. Zenodo. 10.5281/ZENODO.1284472

[evl3304-bib-0040] Machado, H. E. , A. O. Bergland , R. Taylor , S. Tilk , E. Behrman , K. Dyer ., et al. (2021). Broad geographic sampling reveals the shared basis and environmental correlates of seasonal adaptation in *Drosophila* . Elife., 10, e67577. 34155971 10.7554/eLife.67577 PMC8248982

[evl3304-bib-0041] McDonald, J. H. (2014). Handbook of biological statistics. 3rd ed. Sparky House Publishing, Baltimore, MD.

[evl3304-bib-0042] Merçot, H. & S. Charlat . (2004). *Wolbachia* infections in *Drosophila melanogaster* and *D. simulans*: polymorphism and levels of cytoplasmic incompatibility. Genet., 120, 51–59. 10.1023/b:gene.0000017629.31383.8f 15088646

[evl3304-bib-0043] Merila, J. & A. P. Hendry . (2014). Climate change, adaptation, and phenotypic plasticity: the problem and the evidence. Evol Appl, 7, 1–14. 24454544 10.1111/eva.12137 PMC3894893

[evl3304-bib-0044] Miller, R. G. (1997). Beyond ANOVA: basics of applied statistics. Chapman and Hall/CRC, Lond.

[evl3304-bib-0045] Obadia, B. , E. S. Keebaugh , R. Yamada , W. B. Ludington & W. W. Ja . (2018). Diet influences host–microbiota associations in *Drosophila* . Proc. Nat. Acad. Sci. U.S.A., 115, E4547–E4548. 10.1073/pnas.1804948115 PMC5960334 29735646

[evl3304-bib-0046] Olazcuaga, L. , N. O. Rode , J. Foucaud , B. Facon , V. Ravigné , A. Ausset , et al. (2019). Oviposition preference and larval performance of *Drosophila suzukii* (Diptera: Drosophilidae), spotted‐wing *Drosophila*: effects of fruit identity and composition. Environ Entomol., 48, 867–881. 31157861 10.1093/ee/nvz062

[evl3304-bib-0047] Olazcuaga, L. , A. Loiseau , H. Parrinello , M. Paris , A. Fraimout , C. Guedot , et al. (2020). A whole‐genome scan for association with invasion success in the fruit fly *Drosophila suzukii* using contrasts of allele frequencies corrected for population structure. Mol. Biol. Evol., 37, 2369–2385. 32302396 10.1093/molbev/msaa098 PMC7403613

[evl3304-bib-0048] Olazcuaga, L. , J. Foucaud , M. Gautier , C. Deschamps , A. Loiseau , N. Leménager , et al. (2021). Adaptation and correlated fitness responses over two time scales in *Drosophila suzukii* populations evolving in different environments. J. Evol. Biol., 34, 1225–1240. 34097795 10.1111/jeb.13878 PMC8457093

[evl3304-bib-0049] Papaj, D. R. & M. D. Rausher . (1983). Chap 3. Individual variation in host location by phytophagous insects. Pp. 77–124. *in* S. Ahmad , ed. Herbivorous insects: host‐seeking behavior and mechanisms. Elsevier, Amsterdam, The Netherlands.

[evl3304-bib-0050] Papaj, D. R. & R. J. Prokopy . (1989). Ecological and evolutionary aspects of learning in phytophagous insects. Annu Rev Entomol., 34, 315–350.

[evl3304-bib-0051] Poyet, M. , V. Le Roux , P. Gibert , A. Meirland , G. Prevost , P. Eslin , et al. (2015). The wide potential trophic niche of the Asiatic fruit fly *Drosophila suzukii*: the key of its invasion success in temperate Europe? PLoS One, 10, e0142785. 26581101 10.1371/journal.pone.0142785 PMC4651357

[evl3304-bib-0052] Price, T. D. , A. Qvarnström & D. E. Irwin . (2003). The role of phenotypic plasticity in driving genetic evolution. Proc. R. Soc. Lond. B Biol. Sci., 270, 1433–1440. 10.1098/rspb.2003.2372 PMC1691402 12965006

[evl3304-bib-0053] R Core Team . (2014). R: a language environment for statistical computing. R Foundation for Statistical Computing, Vienna.

[evl3304-bib-0054] Rago, A. , K. Kouvaris , T. Uller & R. Watson . (2019). How adaptive plasticity evolves when selected against. PLoS Comput. Biol., 15, e1006260. 30849069 10.1371/journal.pcbi.1006260 PMC6426268

[evl3304-bib-0055] Rausher, M. D. (1988). Plant‐insect interfaces. Ecolog., 69, 295–296.

[evl3304-bib-0056] Ravigné, V. , U. Dieckmann & I. Olivieri . (2009). Live where you thrive: joint evolution of habitat choice and local adaptation facilitates specialization and promotes diversity. Am. Nat., 174, E141–E169. 19737113 10.1086/605369

[evl3304-bib-0057] Reznick, D. N. & C. K. Ghalambor . (2005). Selection in nature: experimental manipulations of natural populations. Integr. Comp. Biol., 45, 456–462. 21676790 10.1093/icb/45.3.456

[evl3304-bib-0058] Rudman, S. M. , S. I. Greenblum , S. Rajpurohit , N. J. Betancourt , J. Hanna & S. Tilk , et al. (2022). Direct observation of adaptive tracking on ecological time scales in Drosophila. Sci., 375, eabj7484. 10.1126/science.abj7484 PMC10684103 35298245

[evl3304-bib-0059] Schmid, B. (1992). Phenotypic variation in plants. Evol. trends in plants, 6, 45–60.

[evl3304-bib-0060] Schou, F. (2013). Fast egg collection method greatly improves randomness of egg sampling in *Drosophila melanogaster* . fly., 7, 44–46. 23247611 10.4161/fly.22758 PMC3660287

[evl3304-bib-0061] Shu, R. , D. A. Hahn , E. Jurkevitch , O. E. Liburd , B. Yuval & A. C. ‐N. Wong . (2021). Sex‐dependent effects of the microbiome on foraging and locomotion in *Drosophila suzukii* . Front Microbiol., 12, 1094. 10.3389/fmicb.2021.656406 PMC8141744 34040592

[evl3304-bib-0062] Shu, R. , L. Uy & A. C. ‐N. Wong . (2022). Nutritional phenotype underlines the performance trade‐offs of *Drosophila suzukii* on different fruit diets. Curr. Res. Insect Sci., 2, 100026. 36003272 10.1016/j.cris.2021.100026 PMC9387456

[evl3304-bib-0079] Stone, H. M. , Erickson, P. A. , & Bergland, A. O. (2019). Phenotypic plasticity, but not adaptive tracking, underlies seasonal variation in post‐cold hardening freeze tolerance of drosophila melanogaster. Ecology and Evolution, 10(1), 217–231. Portico. 10.1002/ece3.5887 31988724 PMC6972814

[evl3304-bib-0076] Tait, G. , Grassi, A. , Pfab, F. , Crava, C. M. , Dalton, D. T. , Magarey, R. , Ometto, L. , Vezzulli, S. , Rossi‐Stacconi, M. V. , Gottardello, A. , Pugliese, A. , Firrao, G. , Walton, V. M. , & Anfora, G. (2018). Large‐scale spatial dynamics of Drosophila suzukii in Trentino, Italy. Journal of Pest Science, 91(4), 1213–1224. 10.1007/s10340-018-0985-x

[evl3304-bib-0077] Tait, G. , Cabianca, A. , Grassi, A. , Pfab, F. , Oppedisano, T. , Puppato, S. , Mazzoni, V. , Anfora, G. , & Walton, V. M. (2020). Drosophila suzukii daily dispersal between distinctly different habitats. Entomologia Generalis, 40(1), 25–37. 10.1127/entomologia/2019/0876

[evl3304-bib-0063] Téfit, M. A. , B. Gillet , P. Joncour , S. Hughes & F. Leulier . (2018). Stable association of a *Drosophila*‐derived microbiota with its animal partner and the nutritional environment throughout a fly population's life cycle. J. Insect Physiol., 106, 2–12. 28916161 10.1016/j.jinsphys.2017.09.003

[evl3304-bib-0064] Tochen, S. , D. T. Dalton , N. Wiman , C. Hamm , P. W. Shearer & V. M. Walton . (2014). Temperature‐related development and population parameters for *Drosophila suzukii* (Diptera: Drosophilidae) on cherry and blueberry. Environ Entomol., 43, 501–510. 24612968 10.1603/EN13200

[evl3304-bib-0065] Torres Dowdall, J. , C. A. Handelsman , E. W. Ruell , S. K. Auer , D. N. Reznick & C. K. Ghalambor . (2012). Fine‐scale local adaptation in life histories along a continuous environmental gradient in Trinidadian guppies. Funct. Ecolog., 26, 616–627.

[evl3304-bib-0066] Tufto, J. (2000). The evolution of plasticity and nonplastic spatial and temporal adaptations in the presence of imperfect environmental cues. Am. Nat., 156, 121–130. 10856196 10.1086/303381

[evl3304-bib-0067] Tufto, J. (2015). Genetic evolution, plasticity, and bet‐hedging as adaptive responses to temporally autocorrelated fluctuating selection: a quantitative genetic model: evolutionary responses to fluctuating selection. Evol., 69, 2034–2049. 10.1111/evo.12716 26140293

[evl3304-bib-0068] Turesson, G. (1922). The genotypical response of the plant species to the habitat. Hereditas, 3, 211–350.

[evl3304-bib-0078] Vacas, S. , Primo, J. , Manclús, J. J. , Montoya, Á. , & Navarro‐Llopis, V. (2019). Survey on Drosophila suzukii Natural Short‐Term Dispersal Capacities Using the Mark‐Release‐Recapture Technique. Insects, 10(9), 268. 10.3390/insects10090268 31450567 PMC6780891

[evl3304-bib-0069] Van Buskirk, J. & U. K. Steiner . (2009). The fitness costs of developmental canalization and plasticity. J. Evol. Biol., 22, 852–860. 19226418 10.1111/j.1420-9101.2009.01685.x

[evl3304-bib-0081] Via, S. (1991). The genetic structure of host plant adaptation in a spatial patchwork: demographic variability among reciprocally transplanted pea aphid clones. Evolution, 45(4), 827–852. Portico. 10.1111/j.1558-5646.1991.tb04353.x 28564044

[evl3304-bib-0070] Via, S. & D. D. J. Hawthorne . (2002). The genetic architecture of ecological specialization: correlated gene effects on host use and habitat choice in pea aphids. Am. Nat., 159, 76–88. 10.1086/338374 18707371

[evl3304-bib-0071] Walsh, D. B. , M. P. Bolda , R. E. Goodhue , A. J. Dreves , J. C. Lee & D. J. Bruck , et al. (2011). *Drosophila suzukii* (Diptera: Drosophilidae): invasive pest of ripening soft fruit expanding its geographic range and damage potential. J Integr Pest Manag., 2, 1–7.

[evl3304-bib-0072] Whitlock, M. C. (1996). The red queen beats the jack‐of‐all‐trades: The limitations on the evolution of phenotypic plasticity and niche breadth. Am. Nat., 148, S65–S77.

[evl3304-bib-0073] Wiman, N. G. , V. M. Walton , D. T. Dalton , G. Anfora , H. J. Burrack & J. C. Chiu , et al. (2014). Integrating temperature‐dependent life table data into a matrix projection model for *Drosophila suzukii* population estimation. PLoS One, 9, e106909. 25192013 10.1371/journal.pone.0106909 PMC4156384

[evl3304-bib-0074] Wood, T. K. , K. J. Tilmon , A. B. Shantz , C. K. Harris & J. Pesek . (1999). The role of host‐plant fidelity in initiating insect race formation. Evolu. Ecol. Res., 317–332.

[evl3304-bib-0075] Yampolsky, L. Y. , T. M. M. Schaer & D. Ebert . (2014). Adaptive phenotypic plasticity and local adaptation for temperature tolerance in freshwater zooplankton. Proc. R. Soc. Bio. Sci., 281, 20132744. 10.1098/rspb.2013.2744 PMC3871322 24352948

